# Modeling daily evapotranspiration time series based on Non-Linear Autoregressive Exogenous (NARX) method and climate variables for a data-deficient region

**DOI:** 10.1371/journal.pone.0318675

**Published:** 2025-02-10

**Authors:** Imee V. Necesito, Junhyeong Lee, Kyunghun Kim, Yujin Kang, Feng Quan, Soojun Kim, Hung Soo Kim

**Affiliations:** 1 Institute of Water Resources System, Inha University, Incheon, South Korea; 2 JHSUSTAIN Inc., Seoul, South Korea; 3 Department of Civil Engineering, Inha University, Incheon, South Korea; UCSI University Kuala Lumpur Campus: UCSI University, MALAYSIA

## Abstract

For flood-prone, developing nations where hydrological data is scarce, an innovative methodological approach is essential. This study aims to explore the potentiality of modelling daily evapotranspiration time series by checking causal relationship among the available climate variables in a flood-prone, data-deficient region like Samar in the Philippines. First, to verify if the available variables (rainfall, air pressure and the four (4) Niño Sea Surface Temperature (SST) Indices) have direct effects to evapotranspiration, a causality test called Convergent Cross-Mapping (CCM) was used. Interestingly, only the Niño SST indices and air pressure were found to have direct effects. Results showed that air pressure and the four (4) Niño SST Indices when combined with Non-Linear Autoregressive Exogenous (NARX) method, can effectively model evapotranspiration. This study raises a significant advancement in evapotranspiration modelling as it is the first to model and pinpoint the potentiality of causal relationship of air pressure and the four (4) Niño SST Indices to daily evapotranspiration time series. This method is found to be potentially suitable for disaster-prone regions where hydrological data is limited.

## Introduction

For disaster-prone, developing countries where data gathering itself is a challenge, the use of readily available hydrological variables as well as AI-based calculation techniques to speed up the process in aiding disaster response is needed. Evapotranspiration (evaporation + transpiration) is the process of evaporating water from leaves through plant transpiration during photosynthesis. We all know that a warmer climate increases evaporation; however, evaporation response is also dependent on the availability of water in the catchment. According to Liu et al. (2021) [1], despite of lack of detection by previous scientists, climate change has altered land evapotranspiration. Condon et al. (2020) [2] have also pointed out that climate warming can shift the balance between the supply of water and its demand which can slow down the plant water stress under certain groundwater conditions.

Evapotranspiration is also a subject of several studies. In fact, SCOPUS (https://www.scopus.com) have recorded a total of 17,113 scientific articles related to evapotranspiration from 1969 to 2022. The studies vary from management studies, remote sensing, and evapotranspiration response to certain environmental or radioactive factors to linear and non-linear modeling. According to the University of Southern Maine [3], the sun’s radiant energy is estimated to increase by about 6% every billion years. The same energy will evaporate the water from the surface of the earth and therefore a big factor for hydrological modeling. The water cycle, the climate and the weather are also dependent to evapotranspiration (www.usgs.gov).

Some studies have attempted to model evapotranspiration. Nagler et al. (2005) [4] used a vegetation index to model evapotranspiration in New Mexico. Abdrabbo et al. (2015) [5] modeled evapotranspiration using Representative Concentration Pathway (RCP) scenarios in Egypt while Jato-Espino et al. (2017) [6] combined cluster analysis with multiple linear regression and Voronoi diagrams to model evapotranspiration.

Several hydrological models also incorporate evapotranspiration as one of their model inputs. Correct model of evapotranspiration is crucial since it is one of the key variables for modeling other hydrological processes and even in fields like irrigation management and agricultural planning. However, the effectiveness of the evapotranspiration modeling techniques varies depending on the meteorological factors used in the techniques, from temperature-based, radiation-based, and combination techniques [7]. Penman-Monteith is one of the oldest approaches to evapotranspiration calculation. The downside is, evapotranspiration can be directly measured using lysimeters [8], and that the measurement can vary from area to region and be entirely reliant on the regional climatic features [9]. Thus, it is shown that empirical formulation is a notable constraint on evapotranspiration estimation. Models with computer assistance have shown notable advancement in the hydrology and water resources areas during the last few decades [10–13]. For the purpose of estimating evapotranspiration based on the available and observed meteorological variables, artificial intelligence (AI) models have been widely used [14–16]. Multivariate climatic variables have been used to model evapotranspiration but to the best of the authors’ knowledge, none have focused on the combination of Niño sea surface temperature indices and air pressure as well as checked the causalities of the variables in consideration.

In terms of machine learning, there are attempts to model evapotranspiration using temperature, wind speed and rainfall. Mobilia and Longobardi (2021) [17] have used conventional methods to model evapotranspiration. Hameed et al. (2021) [18] have used regression and machine learning techniques in combination with temperature, humidity, and wind speed. However, neural networks became the main approach of researchers like Granata and Di Nunno (2021) [19], Ogunrinde et al. (2020) [20] and Deo and Şahin (2015) [21]. Some have used sea surface temperatures as dependent variables but none have yet used site-specific air pressure in combination with global SST data as well as verified the causation using causality test for variable selection. Among these studies, the most common test of performance involves the use of RMSE and R-squared.

SST stands for "Sea Surface Temperature" as defined by the US Environmental Protection Agency (EPA) (https://www.epa.gov/climate-indicators/climate-changeindicators-sea-surface-temperature), it is the temperature of the ocean surface. In general, these indices are used to track temperature changes based on the average Pacific anomalies.

The National Oceanic and Atmospheric Administration (NOAA) [22] states that when a La Niña event occurs, the trade winds are stronger, which can cause more warm water to be pushed toward Asia and more cold water to be upwelled (raised to the surface) along the west coast of the Americas. On the other side, weak trade winds accompany El Niño episodes, which cause warm water to be pushed back east toward the west coast of the Americas. Based on the Climate Data Guide of the National Center for Atmospheric Research (NCAR) (NCAR, 2018) [23], the following is a definition of the Niño Indices and its respective areas (longitude, latitude):

- Niño 1+2 (0-10S, 90W-80W): The smallest and most eastern of the Niño SST areas, which include the South American coast where El Niño was originally noticed by the locals.
- Niño 3 (5N-5S, 150W-90W): This area used to be the main focus for tracking and forecasting El Niño. It was eventually discovered, though, that Niño 3.4 and ONI are more appropriate for describing El Niño and La Niña occurrences.
- Niño 3.4 (5N-5S, 170W-120W): From the dateline to the South American coast, its anomalies match the typical equatorial SSTs in the Pacific. El Niño or La Niña occurrences are reported to happen when the Niño 3.4 SSTs are more than +/- 0.4 C for six months or longer.
- Niño 4 (5N-5S, 160E-150W): The center equatorial Pacific is where its anomalies are correlated. Compared to the other Niño areas, this one seems to have less variation. El Niño—Southern Oscillation (ENSO) status was determined using changes in SST [24] S1 Fig [25] shows an approximate location of the Niño SSTs.

On the other hand, for most stations, the true barometric pressure of a location is also measured. As defined by the National Weather Service (NWS) (www.weather.gov), atmospheric air pressure is the “pressure exerted by the atmosphere at a point as a result of gravity acting upon the cage or column of air that lies directly above the point.” Since evaporation is the process where liquid water turns into water vapor, and transpiration is the water movement process through plant surfaces, evapotranspiration is the combination of water surface and soil moisture evaporation as well as plant transpiration [26]. According to National Geographic, air pressure has an effect on evaporation. In cases where air pressure is high on the surface of a body of water, the evaporation process will not be easy due to the pressure pushing down the water so it will not leave as a vapor. In this study, upon proving the causation link of air or atmospheric pressure to evapotranspiration, we will use it to simulate the amount of evapotranspiration.

El Nino Southern Oscillation (ENSO), a periodic fluctuation in sea surface temperature and air pressure in the equatorial Pacific, has been linked to stream flow variability [27, 28] in South America, and even East and Southeastern Asia [28] where most developing countries like the Philippines and highly-developed countries like South Korea [29] are located. Hydrological variables like evapotranspiration are rarely a subject of prediction or modeling. In fact, the investigation of the association of variables involving ENSO like Niño Sea surface temperatures (SST) indices and air pressure was not a focus in evapotranspiration, unlike the aforementioned studies in streamflow variability [27, 28].

Many studies tried to make evapotranspiration models. In fact, Pelosi et al. (2020) [30] used air temperature and crop parameters such as Leaf Ara Index (LAI) and albedo to model crop evapotranspiration. Jerszurki et al. (2019) [31] did a sensitivity analysis of Penman-Monteith evapotranspiration by obtaining the sensitivity coefficient for several climatological variables such as air temperatures, solar radiation, wind speed, and vapor pressure. Some have used the available and observed meteorological variables and artificial intelligence (AI) models to model evapotranspiration [14–15, 32].

Additionally, various studies have explored evapotranspiration modeling, particularly machine learning algorithms and techniques. In the paper of Torres et al. (2011) [33], evapotranspiration was modeled using two distinct approaches, a direct approach utilizing conventional calculation methods with daily minimum and maximum air temperatures, and an indirect approach that applied machine learning to forecast air temperatures first before calculating evapotranspiration. Their findings indicated that the indirect, machine learning-based approach provided superior results compared to traditional methods, highlighting the potential of integrating machine learning in hydrological models.

Gocic´ et al. (2015) [34] also tried to forecast evapotranspiration using different algorithms but later concluded that the combination of support vector machine (SVM) and wavelet transform is superior among other earlier developed models. Ferreira and de Cunha (2020) [35] also compared various algorithms and concluded that the deep learning models used in the study (CNN-LSTM) achieved higher performance. Both studies emphasize the benefits of combining different machine learning techniques in forecasting hydrological data.

Lastly, Granata et al. (2024) [36] presented two novel algorithms, the Multilayer Perceptron-Random Forest (MLP-RF) Stacked Model and Correlated Nystrom Views (XNV) to forecast evapotranspiration. The study emphasized that the use of MLP-RF stacked model have outperformed the other algorithms presented. While these studies have significantly advanced the field of evapotranspiration modeling, there is no focus on data-deficient areas and none of the studies have examined the causal relationships among the climate variables used (air temperatures, humidity etc.) and none used Niño Sea Surface Temperature (SST) indices and air pressure, which are particularly relevant for regions affected by El Niño and other climatic phenomena. These variables can have significant, yet complex influences on evapotranspiration, especially in tropical and subtropical climates.

Compared to the studies by Torres et al. (2011) [33], Gocić et al. (2015) [34], Ferreira and de Cunha (2020) [35], and Granata et al. (2024) [36], which primarily focus on the predictive performance of various machine learning models, our study emphasizes the importance of understanding causal relationships among climatological variables. This focus not only enhances model accuracy in predicting evapotranspiration but also provides a more robust framework for application in regions with limited data availability.

Furthermore, the approach of using CCM-NARX, which is specifically designed to work with limited and causal input data, sets this study apart. It offers a practical solution for developing countries like the Philippines, where the collection of comprehensive climate data may not be feasible, yet effective water resource management and disaster preparedness remain critical.

By addressing these specific gaps and proposing a methodology adaptable to other data-deficient regions, this study contributes to a broader understanding and application of evapotranspiration modeling, which enhances the global relevance and impact of hydrological research.

To the best of the knowledge of the authors, only a handful of studies tried to model evapotranspiration using Niño sea surface temperatures (SST) and no one have used air pressure in combination with SST, especially testing the causalities using methods such as CCM for evapotranspiration model.

## Materials and methods

### Convergent cross-mapping

The search for interactions and causal chains between variables in complex systems is extremely important in evidence-based research. Although a correlation link does not guarantee or imply causation, causation might be inferred from it. This is the importance of using a causality test especially to verify causalities in the limited variables available in certain areas (e.g. disaster-prone, developing nations such as the Philippines). This study used Convergent Cross-Mapping (CCM) to show the effects of the chosen climatological variables namely the (4) Niño Sea Surface Temperatures (SST) indices, air pressure, and rainfall. CCM, in simpler terms, is a method that can detect if two variables are affecting each other even if the connection between the two variables are not obvious.

According to Granger (1969) [37], the causality test known as Granger Causality (GC) is better suited for stochastic and linear systems [34]. In systems where GC is unsuitable, such as non-separable systems or systems where the predictability of one variable *Y* is not independently distinct to another variable under consideration, CCM can be employed, as noted by Sugihara et al. (2012) [38]. As a result, CCM is a better method for dynamic systems as it can tell shared variables from system interactions. As he explains in his research, CCM can test causation for dynamic systems that are not completely random and can tell when two states are related. Sugihara et al. (2012) [38] also added that the length of time series is a big factor since those with longer length, *L*, the higher the estimate accuracy of CCM could be.

Sugihara et al. (2012) [38] described CCM further by using the Lorenz system with two shadow manifolds (or low-dimensional representations of the complete system) *M*_*x*_ and *M*_*y*_ that were created using lagged-coordinate embedding *(τ = lag)*. It was mentioned that the shadow manifolds will be significantly denser due to the larger library size or time series length, *L*, which results in a more accurate estimate. The lag time can be chosen at random. With the use of their respective time series data, we employed CCM in this work to link evapotranspiration, the four (4) Niño Sea Surface Temperatures (SST) indices, air pressure, and rainfall. The manifolds, where *τ = lag*, are represented by Eqs (1) and (2) after the application of CCM.


$$M_{x}:x(t)=[X(t),X(t-\tau ),X(t-2\tau )\ldots X(t-(E-1)\tau )]$$ (1)

$$M_{y}:y(t)=[Y(t),Y(t-\tau ),Y(t-2\tau )\ldots Y(t-(E-1)\tau )]$$ (2)

We can designate *m(t)* as a point in the manifold, M, and X as an observation function in some temporal flow, as explained by Sugihara et al. (2012) [38]. There is a time series that corresponds to each function, *X*, that we may denote as *X = X(1)*… .… .*X(L)* in order to monitor or keep track the points within the manifold, *M* (*L =* length of the time series).

At the same time, if we consider *x(t)* as points in the manifold, say *M*_*x*_, where *M*_*x*_ was created using the time-lagged values of X, and if *x(t)* consists of vectors like *X(t)*, *X(t-τ)*, *X(T-2τ* up to *X(t-(E-1)τ)*, with *E* as the dimensional state space and as the lag time, we may argue that *x(t)* on *M*_*x*_ can map *m(t)* on *M*, because *M*_*x*_ and *M*_*y*_ are diffeomorphic reconstructions of their shared manifold, *M*. Thus, two dynamically connected variables, such as *X* and *Y*, can finally map onto one another. As a result, it is reasonable to anticipate a rising correlation as *L* or the size of the library grows. In this study, causal-ccm package in python by Javier (2021) [39] was used for CCM analysis.

The authors have used simplex projection to determine the optimal embedding dimension and followed Taken’s Theorem, which says that an access to variables of a dynamical system is equivalent to having one variable among the accessed variables sampled at sufficiently different time points. Eqs 1 and 2 simply represents the manifolds and its data points and not necessarily the embedding dimension.

### Time series models and NARX model

A Non-linear Autoregressive (NAR) model is a time-series model that converts input sequences into outputs by using both recent and historical samples from a time-series. A neural network model is not required for a non-linear autoregressive model. Furthermore, a non-linear function, which can be neural networks or any other polynomial, is needed for a NAR model’s training. As hinted by its name, NARX, short for Non-linear Autoregressive Exogenous Model [40], benefits from exogenous inputs. Yu et al. (2019) [41] concluded that NARX is a better and an effective method when it comes to forecasting precision compared to other methods (e.g. SARIMA).

For NARX, the following model structure was used in modeling evapotranspiration:

$$ŷ(t+N)=F\left(\begin{array}{c} ŷ(t),ŷ(t-1),\ldots ,ŷ(t-n_{y}),\\ x(t+1),x(t),x(t-1),\ldots ,x(t-n_{x}) \end{array}\right)$$ (3)

Using the previous values of a given time series and another such as *x(t)*, the ancestor series, ŷ*(t)* can be forecasted using the NARX model [41]. The number of instances is ŷ*(t+N)* at time, *t*, and the number of days in lead is *(N)*. While *x(t)* is the independent variable input at time, *t*, (including the Niño SST index and air pressure), *x(t-n*_*x*_*)* is the independent variable input *n* days earlier.

This study used the following NARX structure (see also S2 Fig):

$$ŷ(t)=F\left(\begin{array}{c} ŷ(t),ŷ(t-1),N\_12(t-1),\\ N\_3(t-1),N\_34(t-1),N\_4(t-1),AP(t-1), \end{array}\right)$$ (4)

where *N_12 = Niño 1+2 index; N_3 = Niño 3 index; N_34 = Niño 3*.*4 index; N_4 = Niño 4 index; AP = air pressure*

In this work, utilizing data from several sub-catchments, we used the *fireTS* module in Python to model evapotranspiration.

### Daily ET generation using NARX model

Random Forest Regressor (RFR) function was used as an initial or base model to build the NARX model. Random forest is known to often give a very good output which is why the authors used RFR as a base model before applying NARX. In this study, the authors used scikit-learn library in python.

As shown in S3 Fig, the random forest regression algorithm runs by constructing a multitude of decision trees and outputs the most optimal base model to be used in NARX. In this study we used *fireTS* module in python. The data split for the training dataset is 70% of the total available datasets and the remaining 30% is for the testing data.

### Data

The datasets for the evapotranspiration model using NARX was divided into training and testing datasets. As recommended, the training datasets used 70% of the total number of daily data while the other 30% was used for the testing dataset. S1 Table shows the descriptive statistics of the observed evapotranspiration. The time-scale used for the entire study is daily. The study areas (S-7, S-8, S-9 and S-10) and date range used in this study are the same as the study areas used in the paper published by Necesito et. al (2023) [25] (see S2 Table).

Datasets for determining the variable inputs for evapotranspiration include different climatological variables like the four (4) Niño Sea Surface Temperatures (SST) indices, air pressure, and rainfall. As mentioned by Necesito et al. (2023) [25], “Philippines is a data-deficient country” which is why the variables like the four (4) Niño Sea Surface Temperatures (SST) indices, air pressure, and rainfall were chosen. These variables can either be accessed through government institution request or by directly obtaining it from the government website. The Niño SST data was obtained in the National Water Service–Climate Prediction Center (https://climatedataguide.ucar.edu/climate-data/nino-sst-indices-nino-12-3-34-4-oni-and-tni) while air pressure, and rainfall data from stations were gathered from both the Department of Science and Technology (DOST) (https://philsensors.asti.dost.gov.ph/) in the Philippines and through data request in the Philippine Atmospheric, Geophysical and Astronomical Services Administration (PAGASA).

**Fig 1:**
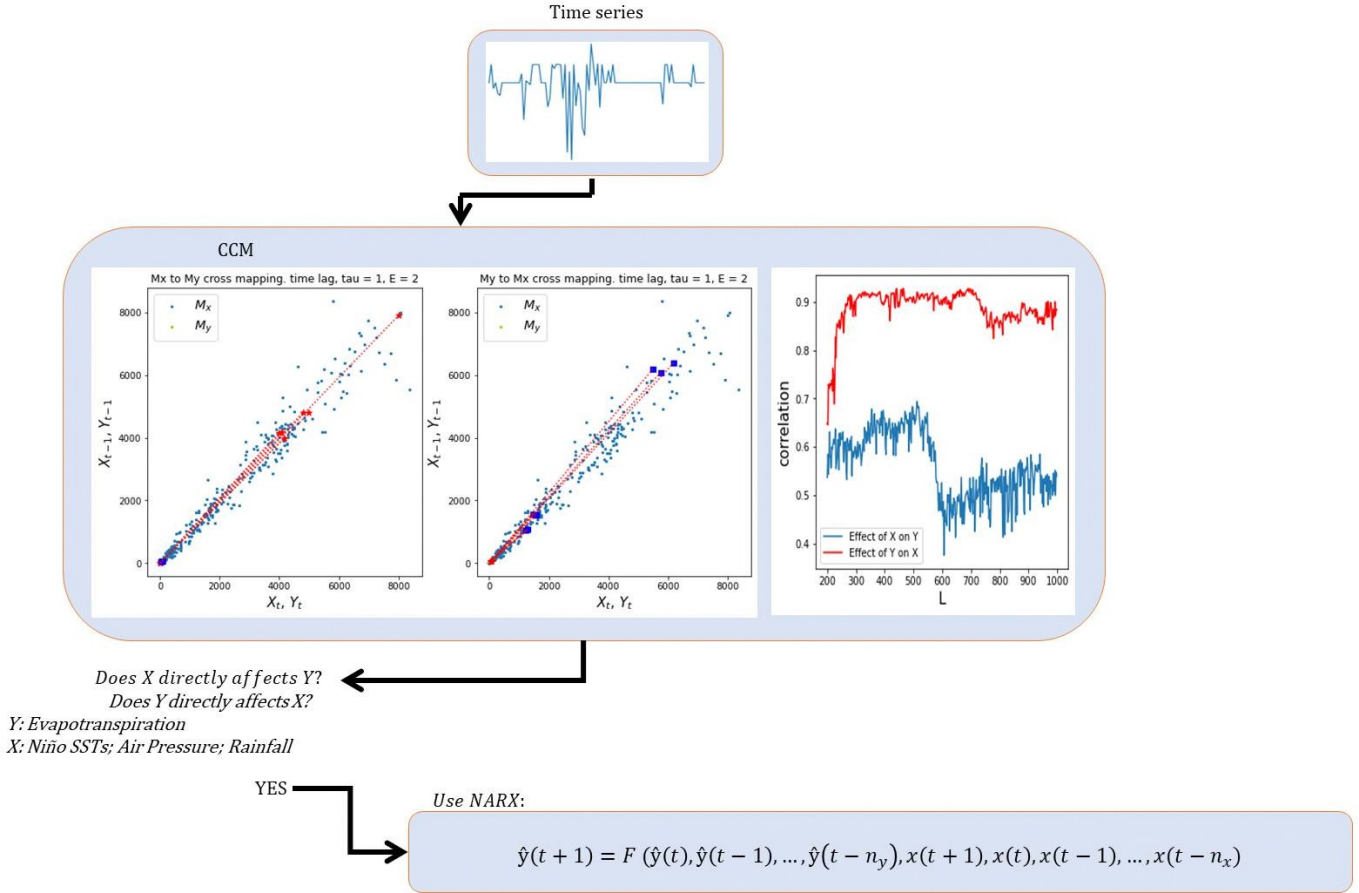
Schematic diagram of the methodology.

A handful of studies have used air pressure as a variable to model evapotranspiration but based on the authors’ knowledge, combining it with Niño SST indices and using NARX to model evapotranspiration with causalities being checked is novel. The methodology is shown in Fig 1.

## Results

**Fig 2:**
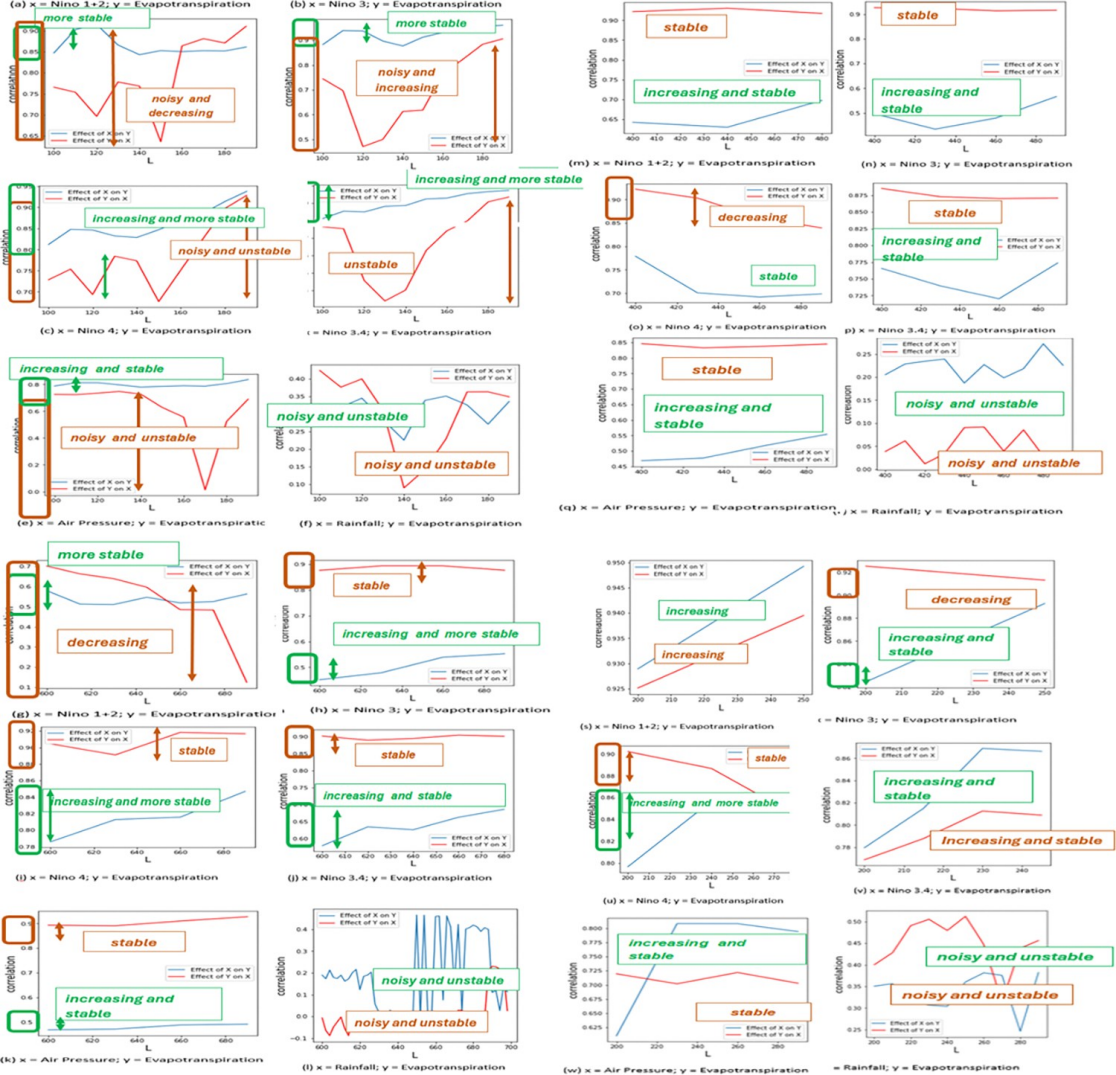
CCM of evapotranspiration and other climatic variables.

This study used CCM plots to detect causation between evapotranspiration and climatological variables such as the four (4) Niño indices (Niño 1+2, Niño 3, Niño4 and Niño 3.4), air pressure, and rainfall. However, as shown in Fig 2, there is a decreasing trend (see red curves in all the plots of (f) evapotranspiration and rainfall.

In CCM, the authors used daily data of all the variables used including the four (4) Niño indices (https://climatedataguide.ucar.edu/climate-data/nino-sst-indices-nino-12-3-34-4-oni-and-tni). It is worth noting that the four (4) Niño indices happened to be available as global monthly data. Each variable (Niño 1+2, Niño 3, Niño4 and Niño 3.4, air pressure and rainfall) was analyzed for direct effect on evapotranspiration.

The authors used CCM to verify the causalities of the initial variables (Niño SST indices, air pressure and rainfall) to evapotranspiration. The variables which have shown a stable and/or increasing trend were chosen as the variables with a direct effect to the evapotranspiration while the unstable one (rainfall (f) in Fig 2) with aggressive up and down trend were not included in the final variable selection.

This paper has used Sugihara’s method for embedding dimension, E, which is simplex projection. Please note that the appearance of instability in the lines or curves is due to the scale and zoom level of the axes, rather than actual fluctuations in the data.

Thus, the apparent instability in the lines (or curves) is a result of zooming in on both the x-axis and y-axis, which exaggerates small fluctuations in the data. In reality, the differences between the x and y values are very small. If we zoomed out, the curves would appear smoother and more stable, showing a consistent increasing trend.

Upon verifying which variables (Niño SST indices and air pressure) have effects on evapotranspiration, the authors proceeded to the NARX model. The authors used a model order of 1 in all the five (5) sub-catchments. By trial and error for order usage while looking at the performance indicator values, the authors arrived at the final evapotranspiration model results which are shown in S4 Fig.

The authors used the variables identified by the Convergent Cross Mapping (CCM) results to validate its recommendations. Performance indicators for each model, corresponding to each sub-catchment (S-7, S-8, S-9, and S-10), were compared, and the final model selection was made based on these performance metrics.

By inspection, most plots found in S4 Fig show almost synchronized peaks and lows of the evapotranspiration amount for the observed and simulated time series which is also reflected in the satisfying values of performance indicators in Table 1. Based on the performance indicators employed, the NARX models for evaporation in the five (5) sub-catchments, (S-7 to S-10) have showed high values of performance indicators. In fact, RMSE, MAPE and RSR are very small in values (near 0.0) which indicate a very good model performance. The same goes with the NSE, IA and LMI which have values near 1.0 which also indicate that the model was able to simulate the values with a high accuracy level and thus, the variables used are effective in modeling the evapotranspiration.

**Table 1:** Performance indicator employed in generating evapotranspiration using NARX model and Niño SST indices and air pressure.

Sub-catchment	MSE	RMSE	NSE	IA	LMI	MAPE	PBIAS	RSR
S-7	0.018	0.133	0.886	0.969	0.991	<0.000	-0.002	0.012
S-8	0.033	0.182	0.758	0.924	0.989	0.004	1.268	0.029
S-9	0.017	0.131	0.890	0.970	0.991	<0.000	0.204	0.012
S-10	0.043	0.207	0.626	0.906	0.987	0.004	1.075	0.035

To strengthen the validation of the method used in this paper, the authors also used SARIMA to compare the differences in the model performances. The results of SARIMA can be found in S3 Table.

Upon comparing the results in Table 1, which used the CCM and NARX models, it is evident that both CCM and NARX significantly outperformed SARIMA across the performance indicators. For metrics like MAPE, RSR, and PBIAS, where values closer to zero indicate better model accuracy, SARIMA exhibited higher values (>0) compared to CCM and NARX across all sub-catchments. Similarly, for performance metrics such as NSE, IA, and LMI, which require values close to 1.0 to signify strong model performance, CCM and NARX consistently outperformed SARIMA. However, both MSE and RMSE values for the models were close to zero, with SARIMA showing slightly lower values than NARX and CCM in some sub-catchments.

Comparing CCM and NARX to SARIMA, it is clear that CCM and NARX outperformed SARIMA.

Despite the outliers shown in S4 Fig, the high peaks and lows of the evapotranspiration were still captured by the simulated evapotranspiration NARX model and all the performance indicators have been consistently high which indicate a very satisfactory performance of NARX with Niño SST indices and air pressure.

For S-7, S-8, S-9 and S-10 in S4 Fig, the simulated peak values (shown in red) have captured the observed peak values (shown in blue). In fact, even the low points of evapotranspiration amount have been captured by the NARX model. However, the simulated evapotranspiration has also showed extreme outliers.

## Discussion

Evapotranspiration calculation is a very crucial part in hydrology. With the worsening climate conditions where drought is foreseen to be a common incidence especially to countries like the Philippines where hydrological data is deficient, scarce or sometimes unreliable, calculating using globally available and easier-to-obtain variables such as Niño Sea Surface Temperatures (SST) indices, rainfall and air pressure would be a significant advancement in the field. The aforementioned variables are said to influence weather patterns and climate but not extensively applied altogether in evapotranspiration modelling. This paper also challenges the belief of the conventional understanding that rainfall has a direct effect on evapotranspiration because as what was shown by CCM, rainfall does not have a direct causal effect on evapotranspiration which is contrary to what was expected. This particular finding is in fact, important, for the way hydrological models are constructed especially to regions where possible complex interactions among climate variables exist.

There are a few studies discussing evapotranspiration models, the potential climatological variables that affect it or other potential methods that can be used to model it. This paper has pointed out that evapotranspiration is a product of the different effects of other climatological variables. With the help of Convergent Cross Mapping (CCM), the authors were able to identify the variables that have direct effect to the value of evapotranspiration in each sub-catchment.

As pointed out by Sugihara et al. (2012) [38], as the library size (*L*) increases, a stable and/or increasing correlation should also be expected. In his paper, he pointed out that in dealing with two dynamically coupled variables, *X* and *Y* for example, with manifolds we can call as *M*_*x*_ and *M*_*y*_, mapping each other is possible since they are a diffeomorphic reconstruction of their common manifold, *M*, and thus an increasing correlation can be expected as its library size (*L*) increases.

Five (5) plots in Fig 2(A)–2(E) where the four (4) Niño indices (Niño 1+2, Niño 3, Niño4 and Niño 3.4) and air pressure were used, showed an increasing and/or much stable trends. Again, this is discussed explicitly in the paper of Sugihara et al. (2012). With these results, only the four (4) Niño indices (Niño 1+2, Niño 3, Niño4 and Niño 3.4) and air pressure have direct effects to evapotranspiration, thus the authors used the said variables in the NARX model to simulate the *y-variable* (evapotranspiration).

The results of CCM have showed that rainfall have no direct effect in evapotranspiration. This is evident when the CCM plots of each sub-catchment of each variable versus evapotranspiration have showed unstable and noisy trend Fig 2(F), 2(I), 2(R) and 2(X) where it should be increasing and/or stable as the library size (*L*) increases. The other five variables including the four (4) Niño indices and air pressure have showed the causality requirement which were pointed out by Sugihara et al. (2012) [38]: increasing and/or stable as the length of the time series increases.

For the NARX model, where the five variables (Niño indices and air pressure) were used as independent variables, the evapotranspiration results based on the performance indicators are generally satisfactory indicating that the NARX model approach using the five (5) chosen variables is effective in simulating evapotranspiration amount.

The outliers in S4 Fig can be attributed to the use of monthly data of the Niño SST indices. According to Fitts (2013) [42], evapotranspiration fluctuations are associated to “variations in the temperature and humidity of the air, with higher evapotranspiration rates on warmer, dryer days”. According to Liu et al. (2022) [43], SSTs have effects on humidity. The same goes with Shie et al. (2006) [44] who emphasized how humidity increases with SST and temperature. The complex association between the climatic variables could be one of the reasons for fluctuating evapotranspiration raw data. However, SSTs are affected by human factors such as pollution or greenhouse gases and even heat energy that comes from the sun (https://www.climate.gov/news-features/understanding-climate/climate-change-ocean-heat-content).

Hanson (1991) [45] also said that the daily fluctuations in evapotranspiration model is also possible due to the rapid increase in the rate of transpiration early in the day and maximum in the mid-day which causes closure to plants’ stomata which decreases transpiration.

Another reason for the presence of outliers in S4 Fig could be attributed to another missing exogenous variable. In this study, due to emphasis on the application on data scarce regions, only air pressure and Niño SST indices were used as variables for the evapotranspiration model. There is a possibility that an addition of one or maybe more variables will reduce the outliers.

Generally, hydrological data by nature, is also composed of stochastic components. This stochasticity adds challenge in forecasting. These challenges include the presence of abnormal events that can potentially create spurious data points resulting in unexpected patterns. Another challenge which is pretty common in regions are outdated instrumentation and operation error (e.g. poor calibration). Therefore, pre-processing of acquired hydrological raw data should be implemented. In fact, Yu et al. (2014) [46] have developed a time series outlier detection method to identify data points that deviate from historical patterns. In their paper, they employed a window-based forecasting method by first identifying a threshold value called prediction confidence interval (PCI). The data points that fall outside of the PCI is then considered to be an anomaly.

The random forest algorithm is designed to mitigate overfitting by employing an ensemble approach. Instead of fitting a model to exact point values, it emphasizes making predictions based on the average or majority outcome derived from multiple decision trees. This approach enhances generalization by averaging predictions across numerous trees, thereby reducing the risk of overfitting to the training data. However, when it comes to outliers, the algorithm tends to estimate or predict data points that are notably different from the observed values. It should also be noted that for non-outliers, the algorithm predicts values by averaging outcomes from similar or closely clustered data points, which potentially helps in maintaining accuracy and robustness. This strategy ensures that predictions are based on representative data clusters rather than isolated anomalies. This could cause widely-observed discrepancies on the predicted model.

The authors plotted S5 Fig (where the DWT+LSTM rainfall model was derived from the published work of Necesito et al. (2023) [25]) to verify the behavior of evapotranspiration and rainfall in all the five (5) sub-catchments. This is also to visually check how evapotranspiration reacts in every increase in rainfall. According to some researchers, findings show that there are varying trends of evapotranspiration due to rainfall effects in different study areas. This is probably the reason why sub-catchments S-8 and S-9 have almost synchronized evapotranspiration trends with rainfall while other sub-catchments have shown opposing rainfall-evapotranspiration trends. It’s also evident that in CCM, rainfall time series in the sub-catchments did not appear to have causality effects (do not have increasing and/or stable trend) to the evapotranspiration time series.

There have been quite a few studies on how evapotranspiration affects rainfall and vice versa especially in the Philippines where reliable data is lacking. Yang et al. (2016) [47] stated that in the case of Loess Plateau in China, the declining trend of evapotranspiration in the area was also attributed to the declining trend of precipitation. However, a different finding by Mishra (2013) [48] suggests that above-normal precipitations which result to cooling of the surrounding results to lower evapotranspiration.

Necesito et al. (2023) [25] have simulated rainfall for the same study areas using Discrete Wavelet Transform and Long-Short Term Memory Network (LSTM). For S-7, the simulated evapotranspiration by NARX model have shown that the evapotranspiration is high on days when there is no rainfall recorded. The peak values of rainfall recorded lowest values of evapotranspiration. Trends of evapotranspiration in S-10 have shown an opposite trend in rainfall despite minimal differences in rainfall values. However, for S-8 and S-9, the increasing trends in evapotranspiration have corresponded to the increasing trends in rainfall which is the same as the findings of Mishra (2013) [48].

The authors believe in the strength of the techniques and methods used. The use of Niño SST indices captures El Niño-Southern Oscillation (ENSO), which has well-documented effects on global weather patterns, including rainfall [49] and droughts [50]. These indices reflect large-scale atmospheric dynamics that can have direct impacts on evapotranspiration, making them particularly useful for modeling in regions where local data might be sparse or unreliable.

On the other hand, air pressure is included in the model variable options since it provides information of atmospheric conditions [51]. Additionally, Penman-Monteith equation for evapotranspiration involves the use of psychometric constant *γ* = (*M*_*a*_/*M*_*w*_)/(*C*_*P*_ ⋅ *P*_*air*_/*λ*), where *M*_*a*_ , *M*_*w*_ and *P*_*air*_ are the molecular mass of dry air, the molecular mass of wet air, and the air pressure, respectively [52].

In more conventional studies performed, evapotranspiration models often rely on local meteorological variables but this study is innovative because it incorporated global climate drivers such as Niño SST indices and air pressure thereby capturing larger atmospheric processes which influence evapotranspiration.

The use of CCM was chosen for its ability to establish causality among variables within complex and nonlinear systems. Unlike traditional correlation analysis, CCM is able to test causal relationships in dynamically coupled systems which is very helpful in understanding the interactions among climate variables and evapotranspiration. Knowing that there is causality among variables being considered is vital in ensuring no irrelevant data is included in the model, thus increasing the accuracy and interpretability of results. Using CCM ensures variables are not simply correlative but actually causative in nature.

NARX, have been proven to be highly effective in predictive modelling [53–55]. While deep learning models such as LSTM is gaining attention in the hydrological field, this study used NARX and global climate drivers—Niño SST indices and air pressure in an evapotranspiration context which, in the authors’ knowledge, has not been explored altogether before.

Combining NARX and CCM is a rigorous two-step approach ensuring only relevant variables were used. This approach has a great potential to be applied in data-scarce regions where data can be unreliable or unavailable. Relying on globally available data such as Niño SST indices and air pressure tends to be a more practical approach for countries or areas where there is limited infrastructure for environmental monitoring and where conventional evapotranspiration models might fail. The aforementioned approach makes the model reliable and robust for climate impact assessment due to careful selection of variables.

## Conclusions

The authors believe that the methodology used, specifically the use of globally-available variables like Niño SST indices and air pressure with NARX, is scalable and can be applied to other areas or regions with similar climatic conditions but with sparse or unreliable climatological data. Thus, the proposed methodology ensures that even in data-deficient regions, accurate and reliable evapotranspiration predictions can be made.

In arid and semi-arid regions, where water resources are often limited and evapotranspiration constitutes a significant component of the water cycle, the model presented in this study could be instrumental in optimizing irrigation schedules, managing water reservoirs, and planning for drought mitigation. By providing reliable estimates of evapotranspiration, even in the absence of extensive local data, the model would be able to support sustainable water resource management. For regions heavily dependent on agriculture, especially where local weather stations are sparse, the ability to predict evapotranspiration using global climate indices can help farmers optimize irrigation practices. This ensures crops receive the right amount of water, potentially increasing yields and contributing to food security in vulnerable areas.

In data-deficient regions, where decisions for irrigation systems and water storage facilities must be made with limited information, the insights provided by this study can serve as a guideline in order for authorities to make more informed and effective planning. Understanding evapotranspiration patterns helps ensure that such infrastructure is appropriately scaled and located to meet the actual water demands of the localities.

For policy-makers in regions with scarce data, this study would be helpful for crafting effective water management policies. By relying on global climate information, which is consistently monitored and updated by global institutes, the model offers a way to develop policies that are responsive to global climatic condition.

While this study focuses on a specific geographic area, the authors believe that the methodology is broadly applicable to any region facing data deficiencies. By utilizing globally available climate information, the methodological approach can be adapted to a wide range of environments. Thus, making it a valuable tool for hydrological modeling and water resource management, globally.

The practical applications discussed demonstrate that the findings of this study have significant implications beyond the analyzed Philippine region. By providing a robust framework for evapotranspiration modeling in data-deficient areas, this study enhances both its scientific impact and its potential to contribute to sustainable water management and agricultural practices in regions facing similar challenges worldwide.

However, since the approach was only applied in, technically, one (1) location in the Philippines (even though tested through different subcatchments), the authors recognize the limits of our findings to regions with different climate profiles. Favorably, the methodology (combining CCM and NARX model) is flexible and can be adapted or extended to other regions. This is due to the fact that CCM served as a filter which can identify which variables could be considered as causative. Thus, by incorporating region-specific climate data, CCM could identify the variables to be included in the NARX model.

To improve the accuracy as well as the relevance of the model, other data sources could also be included (soil moisture, landuse or vegetation cover). For example, in arid or desertic areas, where evapotranspiration could be highly influenced by soil moisture or vegetation cover, analysis should include the aforementioned data (soil moisture or vegetation cover).

In general, conducting comparative studies across diverse environmental conditions (coastal, mountainous, and/or arid environments) could provide more insights in the model performance necessary for broader applicability.

The results demonstrate how deep learning, NARX specifically, can capture and model evapotranspiration by using several climatological variables such as the four (4) Niño SST indices (Niño 1+2, Niño 3, Niño4 and Niño 3.4) and air pressure. This a breakthrough in the field of hydrology because to the best of the authors’ knowledge, there are no studies which tried to combine Niño SST indices to air pressure, nor use air pressure alone or Niño SST indices alone to model evapotranspiration while checking the causalities among the variables. With the use of NARX and as evaluated by the different metrics used, with causality support from CCM, the authors were able to model evapotranspiration. Again, the authors find this beneficial especially for countries whose climate or hydrological data are very scarce.

However, the model’s accuracy could be affected by monthly Niño SST indices data. Future research could explore the use of higher temporal resolution data such as a weekly Niño SST indices data in order to capture the short-term variations in evapotranspiration.

Another limitation of this study is that it focused only in a single climatic region, a tropical, data-sparsed country. Future studies could apply the proposed methodology in diverse geographical settings (e.g. arid, semi-arid or other tropical regions). The authors believe that this would help evaluate the robustness and other area-specific factors that may be influencing the evapotranspiration dynamics.

The NARX model, while found to be effective, may have limitations in capturing a more complex and dynamic interaction of climatic variables. Thus, future studies could focus on integrating more advanced machine learning techniques to improve the model’s predictive accuracy. The authors also suggest that incorporating variables such as land use changes and other environmental factors, urbanization or other anthropogenic factors would give significant enhancement to the model’s predictive performance. Conducting simulations using different climate change scenarios is another option to advance this study further.

By addressing the limitations of the current study, the authors believe that the future work will help advance the general understanding of evapotranspiration which will help in effective water resources management not just for data-sparse countries worldwide.

The authors also acknowledge that working with sparse datasets may increase the uncertainty of the model. Due to these aforementioned limitations, the authors would like to propose strategies such as mean imputation, regression imputation or by using Multivariate Imputation by Chained Equations (MICE). Future research works may also explore the effectiveness of the imputation methods in combination to the proposed methodology of this paper.

Another proposed strategy to address the issue of limited data and data scarcity is the use of synthetic data points based on the statistical properties of the data on-hand. Thus, data imputation and data augmentation techniques can help in addressing the problem of scarce and sparse datasets.

## Supporting information

S1 Fig Niño SST indices regions (Necesito et al., 2022) [25]. (PNG)

S2 Fig NARX mechanism. (TIF)

S3 Fig Schematic diagram of random forest algorithm. (TIF)

S4 Fig Simulated evapotranspiration using NARX model and Niño SST indices and air pressure (S-7 to S10). (TIF)

S5 Fig Rainfall and evapotranspiration plot (S-7 to S-10). (TIF)

S1 Table Sub-catchment name and date range (daily) used (Necesito et. al, 2023). (DOCX)

S2 Table Descriptive statistics of observed evapotranspiration (mm/day). (DOCX)

S3 Table Performance indicator employed in generating evapotranspiration using SARIMA. (DOCX)
