# Supplementary figures and images for: Modeling daily evapotranspiration time series based on Non-Linear Autoregressive Exogenous (NARX) method and climate variables for a data-deficient region
Source: PLoS One. 2025 Feb 10;20(2):e0318675. doi: 10.1371/journal.pone.0318675 (PMC11809863; doi:10.1371/journal.pone.0318675)

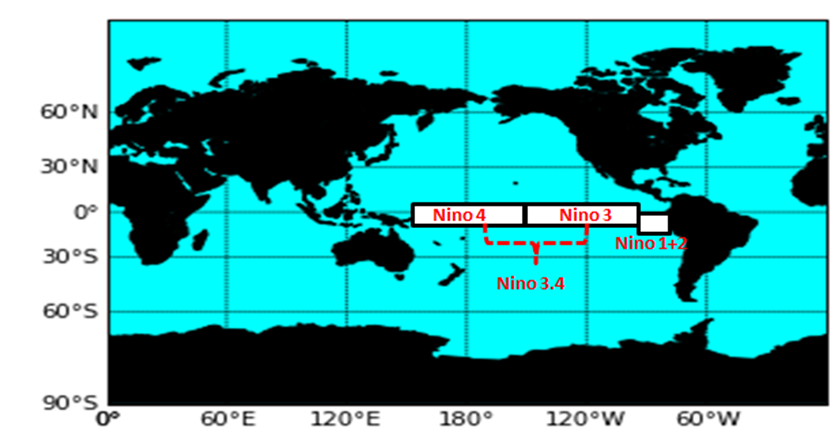

Supplement: S1 Fig — (PNG) [file pone.0318675.s001.PNG]

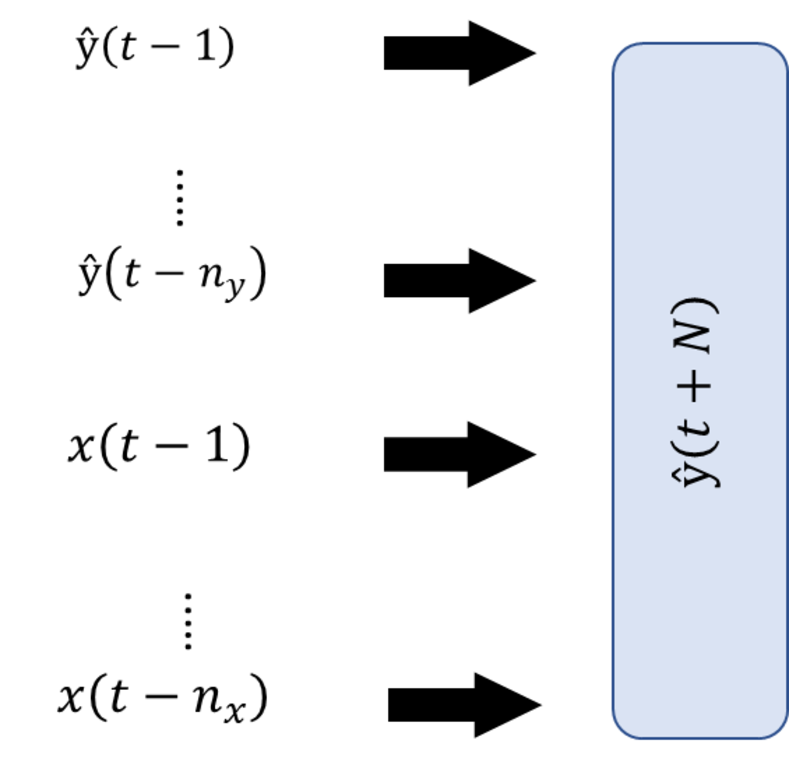

Supplement: S2 Fig — (TIF) [file pone.0318675.s002.tif]

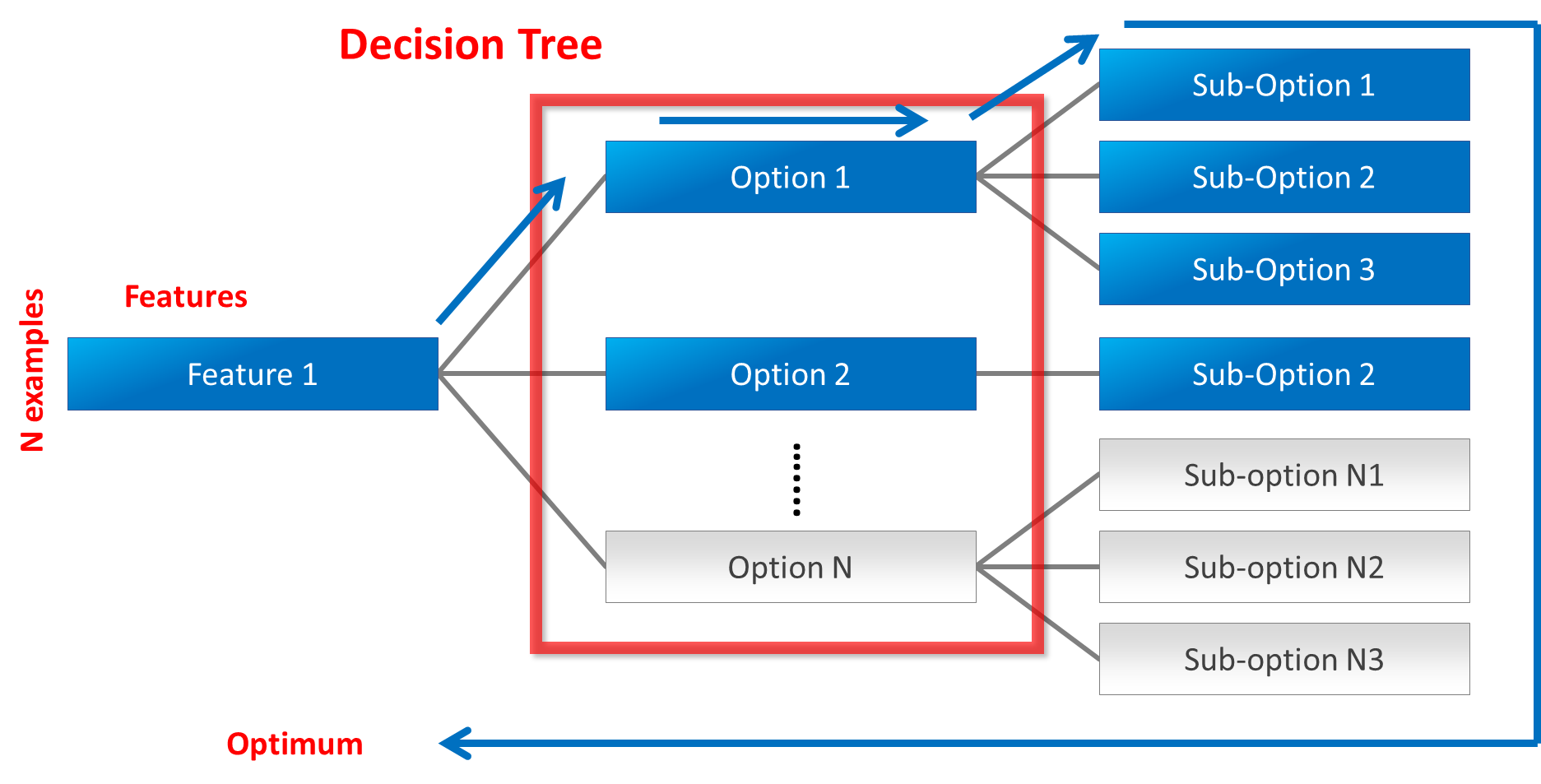

Supplement: S3 Fig — (TIF) [file pone.0318675.s003.tif]

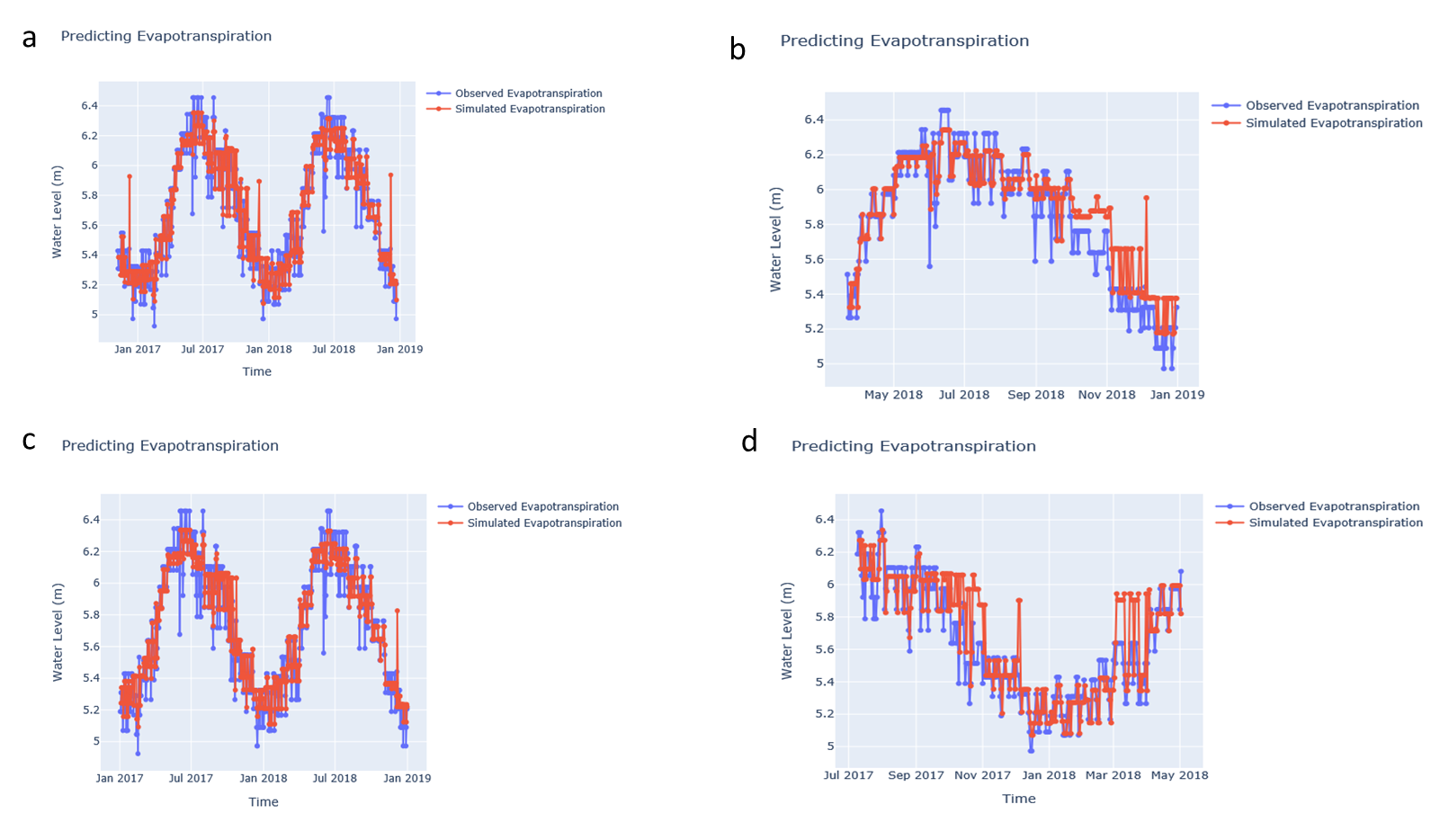

Supplement: S4 Fig — (TIF) [file pone.0318675.s004.tif]

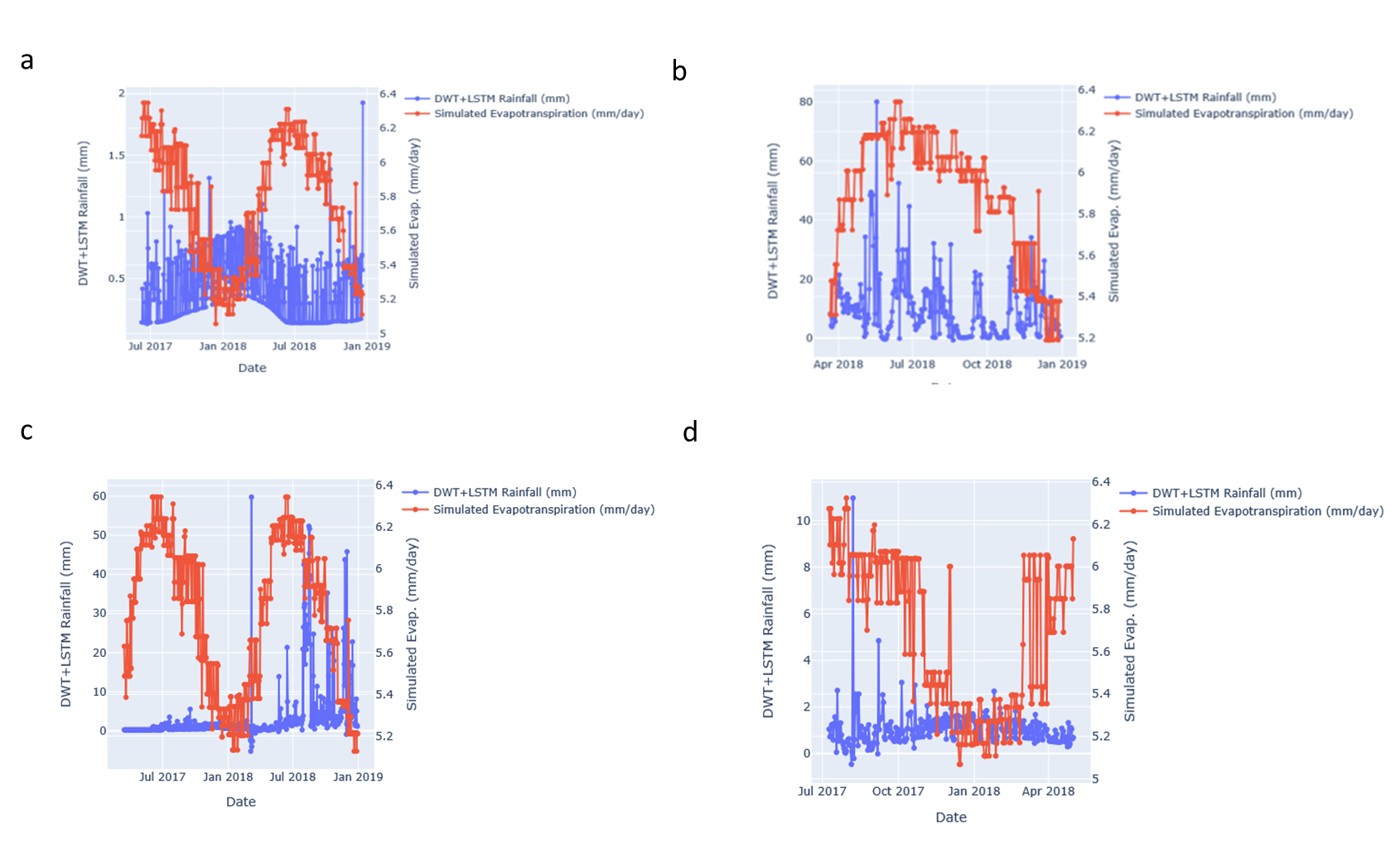

Supplement: S5 Fig — (TIF) [file pone.0318675.s005.tif]
