# Supplementary material for: Modeling daily evapotranspiration time series based on Non-Linear Autoregressive Exogenous (NARX) method and climate variables for a data-deficient region
Source: PLoS One. 2025 Feb 10;20(2):e0318675. doi: 10.1371/journal.pone.0318675 (PMC11809863; doi:10.1371/journal.pone.0318675)
Supplement: S1 Table — (DOCX) [file pone.0318675.s006.docx]

<S1 Table>. Sub-catchment Name and Date Range (Daily) Used (Necesito et. al, 2023)

| Sub-catchment Name | Station Number | From | To |
| --- | --- | --- | --- |
| S-7 (Oras) | 1155 | 6 November 2013 | 22 December 2018 |
| S-8 (Dolores) | 1767 | 22 March 2016 | 31 December 2018 |
| S-9 (Can-avid) | 93 | 2 January 2013 | 31 December 2018 |
| S-10 (Catubig) | 547 | 9 July 2013 | 2 May 2018 |
