# Supplementary material for: Modeling daily evapotranspiration time series based on Non-Linear Autoregressive Exogenous (NARX) method and climate variables for a data-deficient region
Source: PLoS One. 2025 Feb 10;20(2):e0318675. doi: 10.1371/journal.pone.0318675 (PMC11809863; doi:10.1371/journal.pone.0318675)
Supplement: S2 Table — (DOCX) [file pone.0318675.s007.docx]

<S2 Table> Descriptive Statistics of Observed Evapotranspiration (mm/day)

|  | S-7 | S-8 | S-9 | S-10 |
| --- | --- | --- | --- | --- |
| count | 1873 | 1015 | 2190 | 1759 |
| mean | 5.74 | 5.78 | 5.75 | 5.73 |
| std | 0.39 | 0.39 | 0.38 | 0.39 |
| min | 4.92 | 4.92 | 4.92 | 4.92 |
| 25% | 5.41 | 5.43 | 5.41 | 5.41 |
| 50% | 5.76 | 5.85 | 5.76 | 5.72 |
| 75% | 6.10 | 6.10 | 6.10 | 6.08 |
| max | 6.46 | 6.46 | 6.46 | 6.46 |

*‘count’ represents the number of data; ‘mean’ of the rainfall data; ‘std’ is the standard deviation; ‘min’ and ‘max’ is the minimum and maximum value; ‘25%’, ‘50%’ and ‘75%’ are the percentile of the data.*
