# Supplementary material for: Modeling daily evapotranspiration time series based on Non-Linear Autoregressive Exogenous (NARX) method and climate variables for a data-deficient region
Source: PLoS One. 2025 Feb 10;20(2):e0318675. doi: 10.1371/journal.pone.0318675 (PMC11809863; doi:10.1371/journal.pone.0318675)
Supplement: S3 Table — (DOCX) [file pone.0318675.s008.docx]

<S3 Table> Performance Indicator Employed in Generating Evapotranspiration using SARIMA

| Sub-catchment | MSE | RMSE | NSE | IA | LMI | MAPE | PBIAS | RSR |
| --- | --- | --- | --- | --- | --- | --- | --- | --- |
| S-7 | 0.041 | 0.203 | -6.857 | 0.078 | -1.738 | 9.208 | -1052.261 | 0.752 |
| S-8 | 0.032 | 0.179 | -5.17 | 0.035 | -1.572 | 23.268 | -155.798 | 0.716 |
| S-9 | 0.041 | 0.201 | -6.821 | 0.079 | -1.764 | 8.931 | -1495.468 | 0.752 |
| S-10 | 0.018 | 0.133 | -1.436 | 0.463 | -0.613 | 28.067 | -147.908 | 0.444 |
